# A cross-sectional retrospective study of SARS-CoV-2 seroprevalence in  domestic cats, dogs and rabbits in Poland

**DOI:** 10.1186/s12917-021-03033-2

**Published:** 2021-10-07

**Authors:** Małgorzata Pomorska-Mól, Hanna Turlewicz-Podbielska, Maciej Gogulski, Jakub J. Ruszkowski, Magdalena Kubiak, Anna Kuriga, Przemysław Barket, Marek Postrzech

**Affiliations:** 1 Department of Preclinical Sciences and Infectious Diseases, Poznan University of Life Sciences, Wołynska 35, 60-637 Poznan, Poland; 2 Department of Animal Anatomy, Faculty of Veterinary Medicine and Animals Sciences, Poznan University of Life Sciences, Wojska Polskiego 71C, 60-625 Poznan, Poland; 3 Department of Internal Medicine and Diagnostics, Faculty of Veterinary Medicine and Animal Sciences, Poznan University of Life Sciences, Wolynska 35, 60-637 Poznan, Poland; 4 Veterinary Clinic Centrum Małych Zwierząt S.C. M. i P. Barket, Przemysl, Poland; 5 Veterinary Clinic Esculap, Deblin, Poland

**Keywords:** Severe acute respiratory syndrome coronavirus 2, Zoonotic coronavirus, Seroprevalence, Cross-sectional survey, Pets

## Abstract

**Background:**

Coronaviruses (CoVs) have long been known to cause infection in domestic and free-living birds and mammals including humans. The zoonotic origin of SARS-CoV-2 and the biological properties of CoVs, including ability to cross interspecies barriers, enable its emergence in populations of various animals, including companion animals (cats, dogs, rabbits) an area requiring further study. To date, several cases of cats and dogs positive for SARS-CoV-2 and/or specific antibodies have been described. The aim of our cross-sectional retrospective study is to determine seroprevalence of SARS-CoV-2 in domestic dog, cat and rabbit population during recent COVID-19 pandemic in Poland.

**Results:**

In total, serum samples from 279 cats and 343 dogs and 29 rabbits were used in the study. The seroprevalence of SARS-CoV-2 in cats and dogs reached 1.79% (95% CI: 0.77 – 4.13) and 1.17% (95% CI 0.45 – 2.96), respectively (*p* ≥ 0.05). Anti- SARS-CoV-2 antibodies were detected in 5 cats (mean S/P% 106 ± 48.23) and 4 dogs (mean S/P% 78.5 ± 16.58). All 29 samples from rabbits were negative for SARS-CoV-2 antibodies. No significant gender or age differences in seroprevalence in dogs and cats (*p* ≥ 0.05) were found. None of the animals with anti-SARS-CoV-2 antibodies displayed respiratory or gastrointestinal signs at the time of sampling.

**Conclusions:**

Our results confirmed previous findings that SARS-CoV-2 infections in companion animals occurs but are not frequent. Future serological testing of large pet population may provide a comprehensive picture of disease dynamics in companion animals.

## Background

Coronaviruses (CoVs) have long been known to cause infections in domestic and free-living birds and mammals including humans [1, 2]. To date, at least five different CoVs are known to infect companion animals (dogs, cats, rabbits), of which three belong to the *Alphacoronavirus* genus (Feline enteric coronavirus - FECV, Feline infectious peritonitis virus -FIPV and Canine coronavirus - CCoV), two to the *Betacoronavirus* genus (Canine respiratory coronavirus – CRCoV, Rabbit Coronavirus HKU14) [1, 3–7]. As a result of the unique mechanism of viral replication, CoVs have a high frequency of recombination, and together with high mutation rates, may allow them to adapt to new hosts [5, 8].

In December 2019, a new CoV, SARS-CoV-2, was identified in China. SARS-CoV-2 in humans causes a disease called COVID-19 [9, 10]. SARS-CoV-2 is the third zoonotic CoV affecting humans following the emergence of SARS-CoV and MERS-CoV [11, 12].

Since December 2019 SARS-CoV-2 infection has been identified in various species including companion animals. SARS-CoV-2 RNA was detected in dogs [13, 14] and cats (wild and domestic) [15–18]. The antibodies specific to SARS-CoV-2 were detected in cats in Italy, the Netherlands, China and Germany [13, 19, 20]. SARS-CoV-2 was also detected in both European and American minks in several countries. It has also been established that human-to-mink and mink-to-human transmission may occur [21, 22]. To date, there is no data confirming the reverse route of SARS-CoV-2 transmission between animal and human for domestic animals i.e. cat, dog, rabbit. There is also no data confirming natural infection of rabbits, however these animals were susceptible to SARS-CoV-2 infections under experimental conditions [6].

The zoonotic origin of SARS-CoV-2 and the biological properties of CoVs, including ability to cross interspecies barriers, make the possibility of its emergence in populations of various animals, including companion animals (cats, dogs, rabbits) is an area requiring further study. At present, there are only a few studies published assessing the seroprevalence of SARS-CoV-2 infection in household pets (cats and dogs) and no published studies evaluating seroprevalence of SARS-CoV-2 in domestic rabbits. Here we present results from a retrospective serological survey of pets (dogs, cats and rabbits) with the use of samples collected during recent pandemic (between June 2020 and February 2021) in Poland.

## Methods

### Samples

**Fig. 1:**
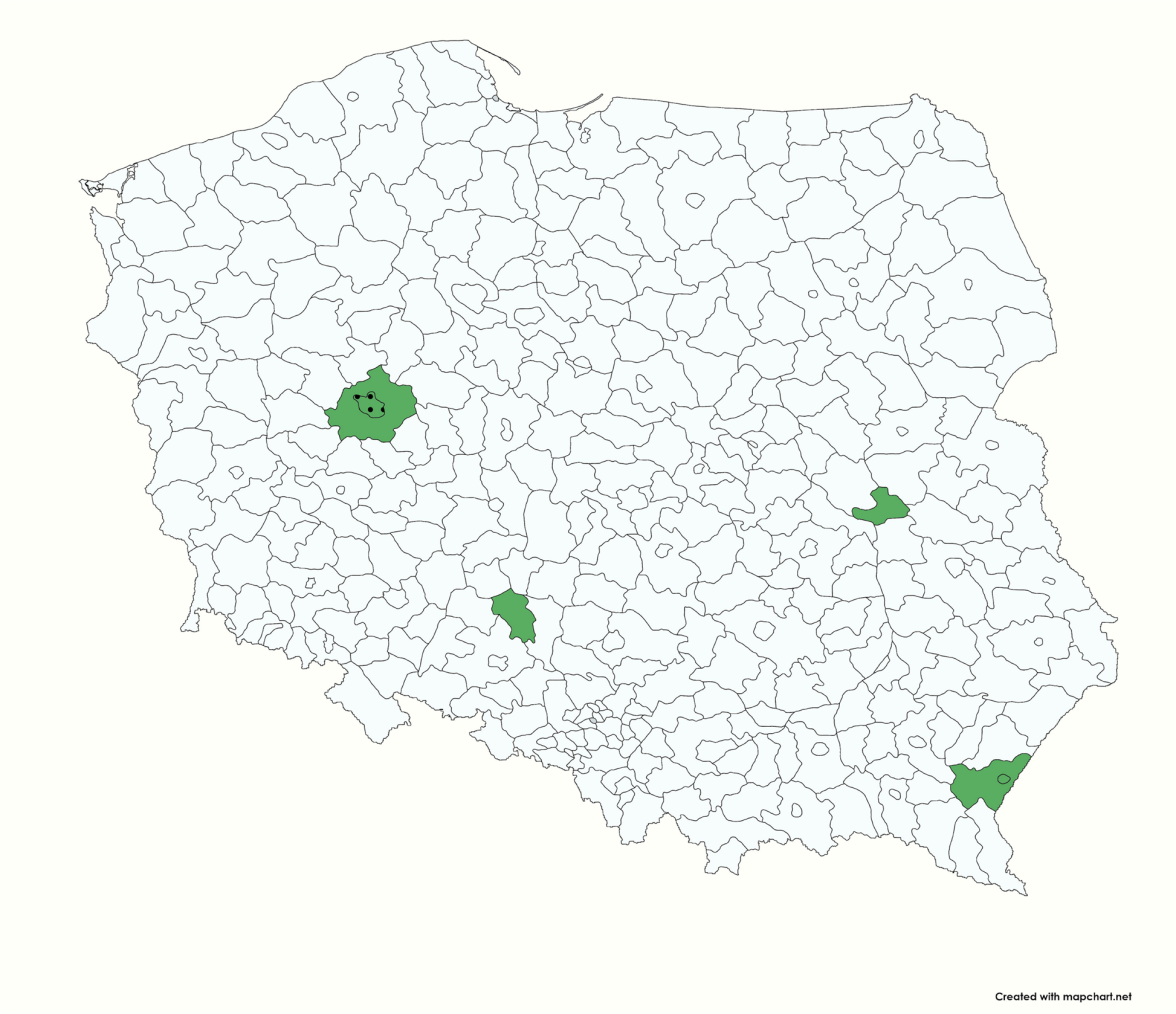
Map of sampling regions. The areas from which the tested samples originated are marked in green. Black dots indicate localisation of practices which submitted samples from rabbits. The detailed data about the sample number per species, date and regions, are presented in Tables 1 and 2

Six hundred and fifty-one serum samples collected in five veterinary practices located in various parts of Poland (Poznan 52°24′24″N 16°55′47″E (wielkopolskie voivodeship); Przemysl 49°47′05″N 22°46′02″E (podkarpackie voivodeship); Kluczbork 18°13′E 50°58′N (opolskie voivodeship); Deblin 21°52′E 51°34′N (lubelskie voivodeship) were selected randomly for this study (Fig. 1). Numbers of samples from cats varied from 11 to 242 per practice, from dogs from 15 to 276 per practice (Tables 1 and 2). Samples from rabbits were collected in one practice (*n* = 29). Sera were stored at − 70 °C in our laboratory until analyses.

**Table 1:** Number of samples from domestic cats that tested positive in the SARS-CoV-2 Double Antigen Multi-species ELISA against the nucleocapsid of severe acute respiratory syndrome coronavirus 2 (SARS-CoV-2) in relation to all samples obtained from domestic cats at a specific time in each location of Poland

Region of Poland (voivodeship)	2020							2021		Total
	June	July	August	September	October	November	December	January	February	
Lubelskie	0/0	0/0	0/0	1/0	10/0	0/0	10/0	0/0	0/0	11/0
Opolskie	0/0	0/0	0/0	0/0	2/0	0/0	0/0	9/0	0/0	11/0
Podkarpackie	0/0	0/0	0/0	8/0	2/0	0/0	4/0	0/0	0/0	15/0
Wielkopolskie	85/2	12/0	0/0	0/0	97/1	0/0	0/0	0/0	48/2	242/5
Total	85/2	12/0	0/0	9/0/0	102/1	0/0	14/0	9/0	48/2	279/5

Number tested/positive

**Table 2:** Number of samples from dogs that tested positive in the SARS-CoV-2 Double Antigen Multi-species ELISA against the nucleocapsid of severe acute respiratory syndrome coronavirus 2 (SARS-CoV-2) in relation to all samples obtained from dogs at a specific time in each location of Poland

Region of Poland (voivodeship)	2020							2021		Total
	June	July	August	September	October	November	December	January	February	
Lubelskie	0/0	0/0	3/0	3/0	0/0	0/0	24/0	0/0	0/0	30/0
Opolskie	0/0	0/0	0/0	0/0	4/0	3/0	0/0	15/2	0/0	22/2
Podkarpackie	0/0	0/0	2/0	3/0	7/0	0/0	2/0	1/0	0/0	15/0
Wielkopolskie	96/0	0/0	0/0	0/0	102/1	0/0	0/0	0/0	78/1	276/2
Total	96/0	0/0	5/0	6/0	113/1	3/0	26/0	16/2	78/1	343/4

Number tested/positive

In total, serum samples from 279 cats and 343 dogs and 29 rabbits were randomly selected and used in the study (Tables 1 and 2). All animals were sampled by veterinary surgeon during a health care visit for various reason (no permission from the Local Ethical Commission was required to collect the specimen). Informed consent for publication of their clinical details was obtained from the owners. For each sample the following information has been available: species, gender, age at sampling, date of sampling, health status at sampling. The samples were collected from June of 2020 to February 2021 (Tables 1 and 2). In addition, a panel of control samples (Table 3) including samples originated from naturally infected cats and dogs with known antibody status against Feline coronavirus virus (FCoV) or Canine respiratory coronavirus (CRCoV), samples from dogs, cats and rabbits collected during 2018 before emergence of SARS-CoV-2 pandemic and archived in our laboratory as well as a panel of commercially available positive human serum samples (COVID-19 positive serum, RayBiotech, USA) were used.

**Table 3:** Set of samples used as a control panel with mean ± SD S/P% values

Control of cross-reactivity			Negative control			Positive control		
Samples positive for antibodies against	Number of samples	Mean S/P% ± SD (range)	Samples collected in 2018	Number of samples	Mean S/P% ± SD (range)	Samples positive for antibodies against SARS-Cov-2	Number of samples	Mean S/P% ± SD (range)
FCoV	7	6.42 ± 15.67 (−7 – 36)	Dog	15	0.73 ± 10.25 (−14 – 19)	COVID-19 positive serum (RayBiotech, USA)	4	81.75 ± 16.58 (68-105)
CRCoV	5	−1.00 ± 11.51 (− 1 – 17)	Cat	17	2.76 ± 11.53 (−12 – 23)			
			Rabbit	14	1.85 ± 8.48 (−12 – 21)			

### Antibody detection

Serum samples from cats, dogs and rabbits as well as positive controls were screened for antibodies against SARS-CoV-2 using the commercial ELISA assay (ID Screen® SARS-CoV-2 Double Antigen Multi-species, IDVet, France), according to manufacturer’s instruction. All samples were assayed in duplicate. The test is dedicated for the detection of antibodies against the nucleocapsid of SARS-CoV-2 in the sera of various species (multispecies ELISA). The assay had a sensitivity and specificity of 100% using 10 positive samples and 47 negative samples, as declared by the manufacturer (Quality control data sheet included in the kit). We obtained similar results (100% sensitivity and specificity) by testing our panel of control sera (Table 3). Positive control intra-plate repeatability ranged from 4 to 7%. Positive control intra-plate reproducibility ranged from 3 to 9%.

Briefly, 25 μl of dilution buffer followed by 25 μl serum sample was added into each well of ELISA plate and incubated at 37 °C for 45 min. The plate was washed three times, then 100 μl HRP-labelled antigen was added into wells at 21 °C for 30 min and the plate was washed three times again. Next, 100 μl of the substrate solution (TMB) was added to each well and incubated at 21 °C for 20 min. As a final step, 100 μl of the Stop solution was added to each well to stop the reaction. The optical density (OD) was measured at 450 nm. For each sample the S/P percentage (S/P%) has been calculated according to following formula:

$$\mathrm{S}/\mathrm{P}\%=\left({\mathrm{OD}}_{\mathrm{sample}}-{\mathrm{OD}}_{\mathrm{NC}}\right)/\left({\mathrm{OD}}_{\mathrm{PC}}-{\mathrm{OD}}_{\mathrm{NC}}\right)\ \mathrm{x}\ 100$$

Samples presenting a S/P%: less than or equal to 50% are considered negative; between 50 and 60% are considered as doubtful and greater than or equal to 60% are considered as positive.

### Statistical analysis

All analyses were performed using a commercial statistical software package (Statistica13.3, TIBCO), with the exception of prevalence (with 95% confidence intervals (CI)), which was available as an online program (https://epitools.ausvet.com.au/ciproportion). Confidence intervals for prevalence were calculated with Wilson score method. Fisher exact test was used for the determination of significance for ordinal and categorical variables. The powers of relationship between the variables were assessed using odds ratios (OR) with their 95% CI. For all tests, *p*-values < 0.05 were considered significant.

## Results

Serum samples from cats, dogs and rabbits as well as positive controls were tested for antibodies directed against the nucleocapsid of SARS-CoV-2 with commercial ELISA. All negative control samples were negative. Four positive controls were positive.

### Cat population

Samples from 20 breeds of cats were used. The most frequent breeds were European Shorthair (196/279), Maine coon and British shorthair (both of 19/279), ragdolls (6/279), Siberian (5/279). For 13 samples from cats no data about breed was available. No relationship between breed and SARS-CoV-2 seropositivity was found. One hundred twenty-four females and 155 males were tested. Sixty out of 279 cats were clinically healthy, 18 revealed respiratory signs, 13 gastrointestinal signs, and 188 had other clinical problems. All cats were divided into four age category: > 1 year (*n* = 32); 1-3 years (*n* = 65), 4 to 7 years (*n* = 62) and ≤ 8 years (*n* = 120). The seroprevalence of SARS-CoV-2 infection in cats reached 1.79% (95% CI: 0.77 – 4.13) and did not differ significantly from the seroprevalence observed in dogs (*p* ≥ 0.05). Number of samples from domestic cats positive against the SARS-CoV-2 nucleocapsid in relation to all samples tested at a specific time in each location of Poland is presented in Table 1. Anti- SARS-CoV-2 antibodies were detected in 5 cats with S/P% ranging from 65 to 183 (mean 106 ± 48.23). In negative samples S/P% ranging from − 14 to 31 (mean 1.37 ± 9.85). None of 32 cats aged < 1 year (0.0%), 2 of 65 aged 1–3 years (6.5%), 1 of 62 aged 4–7 years (2.9%), and 2 of 120 aged ≥8 years (3.0%) tested positive and these differences were no significant (*p* = 0.917) (Table 3). None of the animals with anti-SARS-CoV-2 antibodies displayed respiratory or gastrointestinal signs at the time of sampling. Two females (1.61%) and 3 males (1.94%) were positive in ELISA, but no significant gender difference in seroprevalence was found between females (2/124, 1.61%) and males (3/155, 1.94%) (OR = 1.24; 95% CI = 0.198 – 7.320, *p* = 1.000) (Table 4).

**Table 4:** Seropositivity among cats and dogs split into risk factor groupings

**Risk factor**	**Dogs**			**Cats**		
	**(+) total**	**% positive**	***p***	**(+) total**	**% positive**	***p***
Health status			1.000			1.000
Respiratory disorders	(0) 15	0.00		(0) 18	0.00	
Gastrointestinal disorders	(0) 29	0.00		(0) 11	0.00	
Other disorders	(3) 230	1.30		(4) 188	2.13	
Clinically healthy	(1) 69	1.45		(1) 60	1.66	
Sex			0.366			1.000
Male	(3) 169	1.77		(3) 124	2.42	
Female	(1) 174	0.57		(2) 155	1.29	
Age (years)			0.186			0.917
< 1	(0) 44	0.00		(0) 32	0.00	
1–3	(2) 52	3.84		(2) 65	3.08	
4–7	(1) 67	1.49		(1) 62	1.61	
8+	(1) 180	0.55		(2) 120	1.66	
Total	(4) 343	1.17		(5) 279	1.79	

*P*-value determined by two-side Fisher exact test

### Dog population

Samples from 75 breeds of dogs were used. The most frequent breeds are mixed-breed dogs (92/343), Yorkshire terrier (36/343), Maltese (16/343), German Shepard dogs (11/343) and Shih-Tzu (11/343). For 27 dogs no data about breed was available. No relationship between breed and SARS-CoV-2 seropositivity was found. One hundred seventy-four females and 169 males were included in the study. Sixty-nine out of 343 dogs were clinically healthy at sampling, 15 revealed respiratory signs, 29 gastrointestinal signs, and 230 were diagnosed with other diseases. None of the dogs with anti-SARS-CoV-2 antibodies displayed respiratory or gastrointestinal signs at the time of sampling. All dogs were divided into four age category: > 1 year (*n* = 44); 1-3 years (*n* = 52), 4 to 7 years (*n* = 67) and ≤ 8 years (*n* = 180). The highest seroprevalence was found in dogs aged from 1 to 3 years (2/52; 3.84%). However, the statistical significance was not observed. Of the total 434 serum samples studied, 4 (1.17%; 95%CI 0.45 – 2.96) were determined positive for SARS-CoV-2 antibodies by ELISA. Number of samples that tested positive against the SARS-CoV-2 nucleocapsid in relation to all samples obtained from dogs at a specific time in each location of Poland is presented in Table 2. Anti- SARS-CoV-2 antibodies were detected in 4 dogs with S/P% ranging from 65 to 101 (mean 78.00 ± 17.09). In negative samples S/P% ranging from − 14 to 29 (mean 1.33 ± 10.29). No significant gender difference in seroprevalence was found between males (1.78%, 3/169) and females (0.57%, 1/174) (OR = 3.127, 95%, CI =0.322–30.360, *p* = 0.366) (Table 4). The S/P% values in seropositive dogs ranged from 66 to 101 (mean 78.5 ± 16.58).

### Rabbit population

Serum samples from 16 females and 13 males of domestic rabbit were included in the study. Thirteen out of 29 rabbits were clinically healthy at sampling, 16 were diagnosed with various pathological conditions but these were not associated with respiratory or gastrointestinal symptoms at the time of sampling. The mean age of rabbits was 4.09 ± 2.85 years. Of the total 29 serum samples studied all were negative for SARS-CoV-2 antibody as determined by ELISA.

## Discussion

New data on SARS-CoV-2 infections in companion animals periodically appear in scientific databases. To date, there have been numerous reports of domestic animals from COVID-19 households that tested positive for SARS-CoV-2 and were presumed to be infected by their owners. Cats or dogs infected with SARS-CoV-2 have been identified in Belgium, Hong Kong, US, France, Spain, Germany, UK, Italy [16, 17, 19, 23–28].

The first retrospective survey of seroprevalence SARS-CoV-2 in the population of dogs, cats and rabbits in Poland revealed a similar frequency (1.17 vs. 1.79%) of serum samples that tested positive for antibodies against SARS-CoV-2 in dogs and cats (*p* ≥ 0.05). These results indicate that the human-to-pets transmission of SARS-CoV-2 probably occurs. In contrast, no seropositive rabbits have been found.

Serum samples positive for FCoV and CRCoV antibodies and samples collected in 2018 and archived in our laboratory (as negative controls) were also included in the present study. All control samples were negative what indicates that cross-reactivity or false positive results are unlikely with the test used in the study. These findings confirmed the results of previous studies which likewise did not find any cross-reactivity between SARS and FCoV [19, 20, 29]. Despite very high prevalence of FCoV in the cat population and the fact that FCoV is highly contagious [30, 31], only 5 positive results were obtained after analysis of 279 serum samples, confirming the low probability of sero-cross-reactivity in the present study.

In the surveillance study in Germany conducted at the early stage of COVID-19 pandemic a lower percentage of seropositive cats has been reported (0.69%, 6/920) [19]. Other studies on cats were conducted with the use of smaller number of samples or with the use of samples collected only from pets lived with SARS-CoV-2 positive households [13, 20, 29]. In Wuhan, 14.7% of domestic cats sampled from January to March 2020 tested positive for antibodies in ELISA [20]. The study conducted in France, with the use of samples collected from cats living with owners diagnosed with COVID-19, the seroprevalence was much higher and reached 23.5%. Regarding dogs, 15.4% (2/13) of seropositivity has been observed in the group of dogs lived with COVID-19+ owners, however samples from only 13 dogs were tested [29]. Considering that in our study serum samples were collected randomly during the various pandemic stages (with lower (and higher incidence of SARS-CoV-2 infection in humans) and without connection to COVID-19 status of their owners, the seroprevalence of SARS-CoV-2 in cats fits well with the results obtained in Germany [19]. Considerably higher seropositivity in cats and dogs was reported by Hamer et al. [32], also investigated samples form cats and dogs living in COVID-19 households. The authors have shown that 18.7% pets had SARS-CoV-2 neutralizing antibodies, including 43.8% cats and 11.9% dogs [32].

During the study period, two main stages of SARS-CoV-2 pandemic can be distinguished with respect to the incidence of human SARS-CoV-2 infections [33]. From June to September 2020, the number of SARS-CoV-2 infections in humans was significantly lower than in the period from October 2020 to February 2021 in all voivodeships (around 10 to 230 new cases per day vs. 700 to 4000 new cases per day). In each of these periods, the highest number of SARS-CoV-2 infection in humans was observed in wielkopolskie voivodeship (from about 40 to 230 new cases during first stage and from 700 to 4000 new cases during the second stage). In the remaining voivodeships (lubelskie, opolskie, and podkarpackie), the incidence of SARS-CoV-2 infection in humans were similar (from 10 to 60 during first stage and from 200 to 1500).

Researchers from Italy analysed the epidemiological situation in the canine and feline population between March and May 2020, in relation to the health status of their owners (households) (COVID-19-positive, not know or negative). They showed that seropositivity among dogs living with COVID-19-positive humans was significantly higher compared to dogs living with owners without confirmed SARS-CoV-2 infection (COVID-19 negative group) (12.8% vs. 1.5%) [13]. For cats, the differences were not statistically significant (4.5% vs. 2.6%). An explanation for the lower correlation between COVID status of humans and seropositivity of their household cats may be differences in the behaviour of the cats and dogs. In the case of dogs, close contact with humans is usually more frequent. In our study no significant differences between seropositivity of cats and dogs has been found, but the status of SARS-Cov-2 infection in the households or animals sampled in the present study was unknown. The mean overall seroprevalence of SARS-CoV-2 in dogs has been much higher in the study of Patterson et al. [13] than in our study (4.81% vs. 1.17%). The reason of this could be the use in the previous study a high number of samples from animals that lived with COVID-19 positive humans (around 25% of samples). In contrast, the overall seropositivity was lower (around 3.17%) in cat population than in dogs even though almost 35% of samples were collected from cats owned by COVID-19 positive patients. The above may also be explained by the cats’ greater reticence in contact with humans. The mean seroprevalence reported in cats by Patterson et al. [13] was also higher than in our study (3.17% vs. 1.79%).

CoVs properties, such as Spike-protein plasticity, suggest that species barriers to infection may be easily crossed [34]. SARS-CoV-2 infected humans have been reported to transmit the virus to animals that are in close contact (reverse-zoonotic transmission) [35]. Thus, pet contamination by infected owners is likely given the numerous opportunities for spillover [29, 34, 36]. Two dogs were tested positive for SARS-CoV-2 in Hong Kong [14]. Analysis of the genomic sequence suggested that these dogs were infected by their owners, who also tested positive for COVID-19 [14]. In addition natural SARS-CoV-2 infection has been reported in other domestic animal species such as cat, ferret and mink [13, 37]. Although there is evidence of human-to-cat transmission [16], current data are not sufficient to definitively confirm the occurrence of SARS-CoV-2 in the domestic cat population as a result of cat-to-cat transmission, although high susceptibility of these animals has been found in experimental settings. It has been shown, that cats are highly susceptible to subclinical infection, with an extended viral shedding, and can transmit the infection to other cats through direct contact or aerosols [14, 38, 39]. In addition, increasing reports of SARS-CoV-2 in animals, especially in felids and mustelids, are of concern for the health of these animals but also for public health and wildlife conservation.

The cross-sectional survey carried out between 22 June and 8 July 2020 among police employees in Poland revealed significantly higher seroprevalence of anti-SARS-CoV-2 antibodies than in cats and dogs in the present study [40]. The positive result was reported in 4.3% of subjects for IgG and in 8.9% of subject for IgA + IgM screening test [40]. The lower prevalence of antibodies in cats and dogs suggests that these animals are accidental victims of the of human-to-animals transmission rather than its reservoir for humans or target species. However, the lack of information about the owners health status (diagnosed or no with COVID-19) represents a certain limitation of the present study.

Considering the results obtained so far, there seems to be no indication to take radical measures with domestic animals (cats, dogs, rabbit). However, pets living in SARS-CoV-2 infected household should be kept indoors or on a leash to reduce the potential risk of virus transmission between susceptible host. In addition, any contact between individuals infected with SARS-CoV-2 and animals should be limited and follow basic hygienic practices due to the potential for human-to-animal transmission [41]. Currently, there is no evidence that cats, dogs or rabbits play a significant role in spreading SARS-CoV-2 to people and based on the scarce information available, the risk of these animals spreading COVID-19 to humans is considered to be low [41]. Our results confirmed previous findings that SARS-CoV-2 infections in companion animals occur, although they are infrequent. Future serological testing of larger domestic animals population (dogs, cats, rabbits and/or others) may support the creation of a comprehensive image of disease pattern changes through a pet population.

## Data Availability

The data used to support the findings of this study are available from the corresponding author upon reasonable request.

## Abbreviations

ACE2: Angiotensin-converting enzyme 2
CCoV: Canine coronavirus
CI: Confidence intervals
COVID-19: Coronavirus Disease 2019
CoVs: Coronaviruses
CRCoV: Canine respiratory coronavirus
FCoV: Feline coronavirus virus
FECV: Feline enteric coronavirus
FIPV: Feline infectious peritonitis virus
IgA: Immunoglobulin A
IgG: Immunoglobulin G
IgM: Immunoglobulin M
OD: Optical density
OR: Odds ratio
SARS-CoV-2: Severe acute respiratory syndrome coronavirus 2
TMB: 3,3′,5,5′-Tetramethylbenzidine
